# Benefits, Facilitators, Barriers, and Strategies to Improve Pesticide Protective Behaviors: Insights from Farmworkers in North Carolina Tobacco Fields

**DOI:** 10.3390/ijerph14070677

**Published:** 2017-06-23

**Authors:** AnnMarie Lee Walton, Catherine E. LePrevost, Laura Linnan, Ana Sanchez-Birkhead, Kathi Mooney

**Affiliations:** 1 School of Nursing, The University of North Carolina at Chapel Hill, Chapel Hill, NC 27599, USA; 2 Department of Applied Ecology, North Carolina State University, Raleigh, NC 27695, USA; celeprev@ncsu.edu; 3 Gillings School of Global Public Health, The University of North Carolina at Chapel Hill, Chapel Hill, NC 27599, USA; linnan@email.unc.edu; 4 College of Nursing, The University of Utah, Salt Lake City, UT 84112, USA; Ana.sanchez-birkhead@nurs.utah.edu (A.S.-B.); Kathi.mooney@nurs.utah.edu (K.M.)

**Keywords:** pesticide protective behaviors, Latino migrant and seasonal farmworkers, benefits of protective behavior, facilitators of protective behavior, barriers to protective behavior, strategies to improve protective behavior, tobacco

## Abstract

Pesticide exposure is associated with deleterious health effects. Prior studies suggest Latino farmworkers perceive little control over their occupational health. Using the Health Belief Model as a theoretical guide, we explored the perceptions of Latino farmworkers working in tobacco in North Carolina (*n* = 72) about benefits and facilitators of pesticide protective behaviors as well as barriers, and strategies to overcome barriers to their use. Interviews were conducted with participants at farmworker housing during non-work time. Qualitative data were analyzed using ATLAS.ti. Farmworkers recognized pesticide protective behaviors as helping them to not get sick and stay healthy. Farmworkers perceived work experience as facilitating protective behaviors. Wetness in the field was the most commonly cited barrier to protective behavior use. To overcome this barrier, farmworkers suggested use of water-resistant outerwear, as well as packing a change of clothes for mid-day, with space and time to change provided by employers. Examination of the efficacy and feasibility of farmworkers’ suggestions for addressing barriers is warranted. Training and behavior modeling by experienced peers may improve behavior adoption and perceived control.

## 1. Introduction

Migrant and seasonal farmworkers perceive little control over their occupational health and safety, including pesticide exposure [1]; however, with training and provision of supplies, farmworkers can employ pesticide protective behaviors (PPBs) to minimize exposures [2,3,4,5,6]. The Worker Protection Standard (WPS) issued in 1992 by the United States Environmental Protection Agency (EPA) provided standards for PPBs—such as washing hands and face before eating and drinking and wearing long pants, socks, shoes, and long-sleeved shirts—and mandated that these behaviors be taught to farmworkers within five days of beginning work in the field and every five years thereafter [7]. Recent revisions to the WPS require annual training and no grace period for training before farmworkers begin working [8].

Several studies of self-reported protective behaviors among farmworkers have found limited adherence to these behaviors [9,10]. Epidemiologic studies, however, have not provided a clear picture of the reasons farmworkers may or may not engage in protective behaviors [10]. Barriers to the use of PPBs reported in prior studies have centered on inadequacy of pesticide training, including the language in which it is offered and the uniformity of training [11]. Training that includes a rationale for carrying out PPBs has been shown to increase farmworkers’ perception of control over exposures [1]. 

Personal factors, such as time to do PPBs and comfort, have been reported as barriers [10,11]. Protective equipment may be used selectively when farmworkers feel a financial pressure to work quickly, especially when they are paid at a piece rate [12], and discomfort can be a symptom of heat distress and dehydration. Also cited as barriers have been underestimated exposure risk [13], employer failure to provide personal protective equipment (PPE) or other supplies [13,14,15], and pressure from employers not to use safety equipment [12]. A statistically significant relationship was found between wearing fewer pesticide protective items at work and implementing fewer protective practices at home (such as washing hands right after work, taking off shoes before entering the home, washing work clothes separately from household clothes) when perceived organizational barriers were high [15]. 

One study reported that farmworkers’ motivation to engage in protective behaviors directly corresponded to the availability of the supplies needed to conduct the behaviors; the same percentage of farmworkers who reported hand washing before eating (42%) reported the availability of hand washing water [16]. The lack of separate water for washing and drinking and inadequate availability of soap and towels (lack of supply provision) have been cited as barriers to washing behaviors in other research [17]. In prior research by the authors, adequate supplies for washing were provided in 67% of observations and utilized by farmworkers 35% of the time that they were provided, demonstrating responsibility on both the part of the employers to provide adequate supplies and the part of the farmworkers to use them [18]. Water perceived to be an inappropriate temperature for washing may also be a barrier [12]. While a recent survey reported that if employers provided gloves and other PPE then farmworkers would use the PPE to protect themselves against the harmful effects of pesticides, farmworkers reported they feared job loss for asking for protective equipment [10]. Researchers reported that farmworkers believe their exposures are largely controlled by employers [14]. 

Farmworkers’ perceptions of the benefits of protection against pesticides and other farm chemicals have also been explored, although to a lesser extent. Farmworkers reported believing that protecting oneself from farm chemicals leads to a healthier life, that it is important to protect children from farm chemicals, and that protective clothing is effective against chemical exposure [15]. 

A variety of theoretical frameworks have been used to explain the likelihood that farmworkers will adopt protective behaviors. One of these is the Health Belief Model (HBM) [19]. At its core, the HBM focuses on adherence to preventive health behaviors. It posits that a person’s likelihood of taking a preventive action, in this case engaging in PPBs, is modified by a host of factors. Ultimately, farmworkers and their employers engage in a mental process of assessing perceived benefits and facilitators of the PPBs, as well as perceived barriers to carrying out the PPBs and strategies to mitigate those barriers, in the course of deciding whether or not to engage in them. The HBM has previously been utilized in understanding farmworker pesticide safety knowledge, and demonstrated that increasing knowledge about pesticide safety both decreased perceived risk from pesticides and increased perceived control over pesticide safety [1]. The aim of the present study is to explore the benefits, facilitators, barriers, and strategies to counter barriers that Latino migrant and seasonal farmworkers identify as impacting the PPBs they practice.

## 2. Methods

### 2.1. Participants

The University of Utah IRB determined this study to be exempt on 17 March 2014 (IRB # 00071986). After obtaining Institutional Review Board exemption, seven farm owners/operators from one of the three largest tobacco production counties in North Carolina were approached about participation in this study. These individuals had previously voluntarily participated in a safety intervention called Certified Safe Farm (CSF) to reduce agricultural injuries, demonstrating their orientation toward safety. Three of the seven farm owners/operators agreed to provide access to their farms so that the researchers could approach the farmworkers working for them. Two of the participating farms employed fewer than 10 workers. At the time of data collection, workers on one of those farms were engaged in weeding shortly after the transplantation of tobacco into the field, and on the second farm, workers were engaged in topping and suckering (the routine crop maintenance procedure in which the tobacco flower is removed) prior to harvest. On a third farm, which employed the greatest number of workers, some topping and suckering was done early during data collection, but the majority of the farmworkers were engaged in harvesting of the tobacco and barning activities. 

Farm owners/operators did not participate in recruitment of their farmworker employees, and farmworkers were informed that participation would not impact their employment. Seventy-two farmworkers were approached, agreed to participate, and provided informed consent. Inclusion criteria for farmworkers were being age 18 years or older, having the ability to speak Spanish or English, and self-identifying as Hispanic/Latino ethnicity. Each participant was given a study identification number, which was different from the participant numbers assigned in this paper, and the requirement of written consent was waived because it was the only source linking the farmworkers’ names to data. All data were collected between May and October 2014. 

### 2.2. Methods

A quasi-qualitative approach was selected to examine farmworkers’ perceived PPB benefits, barriers, facilitators, and strategies to mitigate barriers. Data were collected through structured interviews which were audio recorded. The structured interviews were conducted using an interview guide and a demographic questionnaire. The structured interviews took an average of 25 min per participant with a range from 10 to 65 min. All but three interviews were conducted by a native Spanish speaker. The first author observed all interviews and conducted three (in Spanish) when the interviewer was unavailable. The interviews were conducted on non-work time (Sunday afternoons) just outside of the homes of the workers (either employer-supplied or farmworker-rented mobile homes) and, in the case of four workers, at the farm on non-work time per their request to avoid having the research team come to their homes. For participation in the study, farmworkers were given a $25 Walmart gift card and a hat donated by a local farmworker service organization. 

The investigators developed an interview guide to assess farmworker demographic characteristics and perceived benefits and barriers of PPB as described in the HBM, as well as strategies to minimize those barriers and facilitators of PPB. To ensure that respondents focused on pesticide-related behaviors mandated to be taught by the WPS, those behaviors were described for respondents. Open ended questions (16) included items related to perceived benefits (e.g., What are the benefits of pesticide protective behaviors?), barriers (e.g., What gets in the way of you doing pesticide protective behaviors?), facilitators (e.g., Which pesticide protective behaviors are easy to do? Why?), and strategies to minimize barriers (e.g., If you could suggest anything to minimize pesticide exposures what would it be? What strategies could make behaviors that are hard to do easier?). While the questions were the focus of analysis for each of these domains, sometimes relevant comments emerged in other sections of the interviews and were also included in the analysis. Two independent Spanish translations of the interview guide were made, and the versions were compared. The interview guide was pilot tested with two farmworkers not participating in the study; they were asked which questions, if any, caused confusion, so questions could be refined prior to the study. 

The interviews were transcribed and translated by the interviewer, a native Spanish speaker from Mexico whose parents were agricultural workers and who grew up close to the farms in the study. The selection of this team member to do translation and transcription was purposeful, as the literature suggests that a translator’s natal language and dialect should be similar to that of the participants to minimize threats to validity [20]. The interviewer understood the research methodology and the fact that her own social position and lived experience influenced her interaction with the data through interview administration, transcription, and translation, and she and the first author had several conversations about the data as it was being collected [21]. For quality assurance, an independent transcription and translation service was used for seven of the transcripts (10% of the data), which were selected at random, so that differences could be explored. The majority of differences in translation were minor and involved the use of slang and dialectical language.

## 3. Analysis

Demographic data were collected and managed using REDCap electronic data capture tools [22]; the REDCap database was password protected. Data were entered by the first author, and 10% of the data entered were checked for quality assurance and found to be 100% correct. Data were then analyzed in SPSS v22 (IBM Corp., Armonk, NY, USA) [23]. Descriptive statistics, including frequencies, were run for all of the demographic variables. 

The first author content analyzed the interviews for factors that were perceived as benefits, barriers, facilitators, and strategies to minimize barriers to PPBs [24]. A priori codes were factors that the investigators expected to emerge as responses (e.g., “time” and “discomfort” for barriers to PPBs) as a result of prior field work and/or familiarity with existing literature. Emergent codes were those that arose throughout the analyses (e.g., “training” as a facilitator of PPBs). Many codes (e.g. “heat”, “wetness”) were found in more than one family (e.g. barriers, strategies to overcome barriers). Coding was done using ATLAS.ti software (Scientific Software Development, Berlin, Germany) [25]. 

While the first author coded, she listened to each audiotape again for context and as a form of quality assurance. When there were discrepancies between the audio recording and the transcript, the audio-recorded version was used. Seven of the transcripts (10% of the data) were selected using a random number generator for the first author and a second coder to code independently. The first author/coder had extensive knowledge of the study and its participants, and the second coder had none. For every response for which the two coders did not agree in their initial codes, a negotiated code was determined through discussion and transcript review [26]. The interrater reliability was 68.4% for the initial coding process and 100% after negotiation. The first author undertook a secondary review and coding of the data after the negotiation of codes [24].

## 4. Results

### 4.1. Participant Characteristics

The 72 participating farmworkers were predominantly male (96%), were from Mexico (97%), and had an average age of 33 years (range 18–68). The majority (90%) were in the United States on a work contract (H2A visa) and had completed no more than a middle-school education (89%). Additional demographic information, including living accommodations and years in agriculture, can be found in Table 1. 

### 4.2. Benefits of Pesticide Protective Behaviors

When farmworkers were asked about the benefits of pesticide protective behaviors, the most commonly shared response was not getting sick (*n* = 24, 33%) or, framed positively, staying healthy (*n* = 8, 11%). Several farmworkers also talked about not getting cancer specifically (*n* = 3): “Protect yourself against illnesses, cancer and any illnesses” (P62). Others described not getting “poisoned” or “intoxicated” from pesticides (*n* = 7): “It works to not get intoxicated and not get illnesses, for our own health” (P99). Some mentioned avoiding getting skin problems (*n* = 6): “One is protected from the small bumps that one gets with the pesticides” (P101). Also mentioned was not getting dizzy (*n* = 4); for example, “They protect you from not getting full of liquid or for it to penetrate and then you get dizzy” (P31). 

Closely related is the concept of protection from exposure in addition to illness (*n* = 22, 31%). One participant stated, “They [PPBs] are really good because they protect you, they protect your eyes, your nose, your respiration. With any contact with pesticides you have to protect yourself” (P25). Another emphasized illness prevention through exposure reduction, “The more you cover yourself, the less risk you run of getting sick because one has more protection” (P76). 

Avoidance of pesticides altogether was described as a benefit of PPBs. Avoidance of pesticides usually centered on staying out of restricted areas (*n* = 18, 25%): “We already know that when they apply the pesticides somewhere, we do not get close” (P17). Relatedly, another benefit included making the pesticides impenetrable (*n* = 3). 

Two farmworkers perceived PPBs as a way to prevent green tobacco sickness (GTS), a form of nicotine poisoning that occurs by dermal absorption of nicotine from tobacco leaves when the plants are wet. One farmworker discussed the use of water-resistant outerwear in the context of nicotine exposure: “It [PPB] covers many things. If you enter without a poncho, the water from the tobacco can get you ill. But with the poncho, it covers you and not a lot falls on you” (P34). 

### 4.3. Facilitators of Pesticide Protective Behavior

Farmworkers were asked which PPBs were easy to do and why they were easy, so as to understand what facilitates these behaviors. Facilitators were also mentioned in response to other questions, and the most common facilitator that emerged across questions was training (*n* = 19, 26%). The video method was discussed in particular: “I don’t know much, but the five years, five seasons that I have come, I have learned from the videos they play for us” (P101). The role the farm owner/operator plays in the context of the videos was also described: “We watch videos and we have to go by those rules, protect ourselves, and the boss tells us to protect ourselves from the pesticides. He showed us [the video] so we can know the rules and protect” (P11).

Farmworkers also reported the belief that protective behaviors were effective (*n* = 16, 22%), as exemplified by this quotation in which the belief was gained from personal experience: “Use long sleeve[s], gloves, boots, plastic, long pants, and a nylon apron that we have. It is easier because the one time that I worked without it, I was sick in the afternoon” (P51). Farmworkers also mentioned behaviors that they perceived as being effective (i.e., gloves, ponchos, rubber boots during harvest, bandanas, mouth coverings, and hats): “Gloves because they protect us more, the poncho because it protects us from the morning dew, and the rubber boots because it protects our feet” (P59). PPBs were also described as easy to do (*n* = 12, 17%): “It is easy because I feel it is easy; it is an everyday thing” (P47).

Several farmworkers talked about their own agricultural experience and specifically watching others in the field as facilitating their behaviors (*n* = 12, 17%). For example, one farmworker stated, “I have years working and I look at the people. The more we observe, the better ideas we have for ourselves” (P5). Farmworkers also described bringing their experiences from their home countries to the workplace as well: “And all those precautions, we have brought them from Mexico, from the same job, agriculture. You have almost your whole life working in it. It would be very illogical not knowing what you need in order to protect yourself; it’s very dangerous” (P13).

Farmworkers also reported that their having what was needed to do the behaviors made them easy (*n* = 12, 17%): “Our boss provides latex or cotton gloves, mouth covering, and glasses. It would be my mistake not putting the gloves, mouth covering, or glasses on” (P13). Also shared as facilitators were obeying orders (*n* = 9), making PPBs habitual (*n* = 6), making one’s health his/her first priority (*n* = 4), the fact that PPBs take no time (*n* = 3), and mechanization that makes working safer than it used to be (*n* = 3). 

### 4.4. Barriers to Pesticide Protective Behavior

When asked directly, “What gets in your way of doing PPBs?” the vast majority of farmworkers said “nothing” initially (*n* = 52, 72%), but barriers arose in response to questions about which PPBs were hard. Wetness in the field was the most commonly described barrier to PPBs (*n* = 26, 37%). Wetness was described as coming from rain, morning dew, the tobacco itself, and sweat. Farmworkers understood that moisture from the tobacco could make them sick: “The water from the tobacco can get you ill” (P34). Another farmworker explained, “When the morning dew falls onto my lips, I feel my tongue numbing, but nothing else” (P73).

Workers reported concern that sweating could increase their likelihood for pesticide poisoning; one (P18) said, “When one starts to sweat, the pores open and the anxiety begins. If you are not careful to bathe or wash your hands before eating something, since you have your pores open, you get dizzy or vomiting because as a worker, you have your pores open because of the sweat.” Sweating was also described as a reason for not wearing long-sleeved, water-resistant outerwear: “When I put that [protective clothing] on, I sweat more, and if I use that, I am going to sweat even more. That is why I don’t use the long-sleeve one” (P59). Protective clothing that had become wet externally from dew or internally from sweating was understood to be less effective. One farmworker stated, “The shirt starts getting wet and that’s how one starts having contact with the pesticide” (P98).

Heat was a major reason (*n* = 24, 33%) not to use all recommended protective clothing. One farmworker reported, “When the tobacco has a lot of water, one puts on the nylon. Once the sun is heating up, we take it off because we cannot bear it” (P8). Another worker described lifting his protective glasses because of the heat: “Sometimes when the sun is really strong, a lot of the times, the glass of the glasses heats up our vision and a lot of the times you pull them up a little, but it is important to have the glasses on in case the wind brings a pesticide back and it can land in your eyes” (P13). Another participant described health and protective clothing use in this way, “I do not put on a mouth covering because the heat is unbearable being there, so one feels like they are suffocating” (P1).

Farmworkers described ‘flojera’, translated as ‘weakness’ or ‘lethargy’ as a common barrier (*n* = 7, 10%). It was usually described in combination with recognition that PPBs are not hard to do: “[I]t [PPB] is nothing one cannot do” (P76). Time was expressed as a barrier by only a small number of farmworkers (*n* = 3). 

### 4.5. Strategies to Improve Pesticide Protective Behaviors

Perhaps most importantly, the participants were asked to identify strategies to improve PPBs. The majority of farmworkers (*n* = 46, 64%) responded initially that either there were no strategies they could suggest or they did not know what to suggest. Additionally, a couple of participants stated that they did not feel authorized to make suggestions: “Well, no, I say that one cannot have an opinion on that or say what to do. One is not capable of that” (P8). However, a number of valuable suggestions emerged from other participants.

The most cited strategy by farmworkers was to have the necessary supplies on hand (*n* = 12, 17%). Having supplies on hand was described as “tak[ing to the field] the necessary equipment, everything of rubber and everything necessary to do those jobs” (P66). More farmworkers spoke to employer provision of supplies as a strategy than spoke of lack of it as a barrier. For example, one participant had this message for employers, “Give us mouth coverings and ponchos; in case one rips, we can change” (P58).

Participants suggested changing one’s clothes during the day (*n* = 7, 10%) both to decrease pesticide exposure and to minimize the reported barrier of wetness. One respondent said, “If one feels bad with the dirty clothes, change against an illness since there are some that get hives and are scratching and scratching all the time. Take a change of clothes to change over there [at the work site]” (P66). Changing clothes at mid-day was specifically mentioned: “Change at noon. I change my shirt and undershirt, and put clean gloves on, shoes” (P57). One person mentioned that the time when transferring from one field to another (which usually required traveling by bus) was a good time to change clothes as well: “Change clothes, because when you change fields, you have more time to change your shirt or something” (P91). Farmworkers described preparing a bag with a change of clothes and supplies (*n* = 7, 10%). “I have an apron, rubber gloves in store and when one needs them, they warn us. We take a bag with clothes, with the gloves and everything one needs in a bag, and there is always a bathroom close by to wash our hands. So one washes and uses what one brought” (P25). Multiple farmworkers described efforts to change clothing throughout the day; however, keeping up with the associated washing of clothing was described as a challenge: “The hardest is that we have to wash our clothes every day, and sometimes in the morning one has to change at mid-day because in the morning one gets wet and then we get full of dirt and we have to change again” (P8). 

Communication of warnings among workers (*n* = 6, 8%) was also mentioned as a strategy: “When they’re spraying and pouring liquid, we try to tell one another, ‘Not in that field.’ One takes care of another” (P94). Only one participant spoke of warnings coming from a supervisor: “Talk to people. Tell them they can’t harvest, or if it’s fruit, not touch it until they tell you. There is someone that gives orders of what can and cannot be touched” (P102). 

Other strategies that were suggested by participants could not be linked to a specific barrier. For instance, farmworkers suggested using medications or ointments (*n* = 8) to counter the adverse effects of pesticides, including skin rashes: “I put itch medication on every day” (P49). Also mentioned were using less or no pesticide (*n* = 5), using “suero” (*n* = 4), and using milk (*n* = 4). “Suero” was the word used to describe a powdered form of electrolytes that was frequently observed by the researchers in use in the field. One respondent explained: “You saw that we carry small packs of ‘suero’ in order to withstand the temperatures, so we won’t dehydrate. With that, our temperature drops and so does the heat. We bring it from Mexico and we also use it over there. Once you drink it, fifteen minutes pass and you are comfortable. It comes out being like a Gatorade. It is a powder that we put in our water. We cut the tobacco here with machines, but over there, it is with the hands, and if you don’t drink it, you’ll just be vomiting. We look for ways to help ourselves and move forward” (P18). Several participants described drinking milk to prevent effects of pesticides: “I drink milk. They say that milk helps to cut the intoxication that is what they say. I drink milk in the morning and in the afternoon as well. Also, at night when I can’t sleep, I drink a glass of milk” (P51). 

## 5. Discussion

Utilizing a quasi-qualitative approach, we examined in their own words farmworkers’ perceived benefits, facilitators, barriers, and strategies to mitigate barriers to pesticide protective behaviors. This approach identified factors that impact farmworkers’ use of pesticide protective behaviors on tobacco farms, and yielded meaningful, actionable suggestions for training and interventions to increase pesticide protective behaviors use. 

### 5.1. Health-Related Benefits of Protective Behaviors

Previous studies have found that farmworkers are more familiar with short-term effects of pesticides than long-term effects [13,27], and our results were consistent with those findings in that farmworkers more frequently identified preventing short- rather than long-term effects of pesticide exposure as benefits of implementing protective behaviors. Consistent with the literature [15], farmworkers described minimizing pesticide poisoning/intoxication, skin problems, and dizziness. Farmworkers did not speak of longer-term risks, such as neuropathy, immune dysfunction, or Parkinson’s disease [28,29,30]. Cancer was mentioned by a few farmworkers, but no farmworkers reported truly understanding the connections that exist between pesticides and cancer [31,32]. Long-term risks, in addition to short-term risks, should be emphasized in pesticide training for farmworkers. 

A benefit of PPBs identified by two farmworkers in this study was protection against green tobacco sickness (GTS) the symptoms of which are similar to pesticide poisoning and heat illness [33]. Other researchers have previously found that farmworkers have difficulty identifying which risks are associated with pesticides and which are associated with contact with the plants themselves [27,34], and our finding is not surprising in tobacco farming, where there are risks for adverse health effects from nicotine, heat, and pesticides. Videos and other training for tobacco workers should emphasize that protective behaviors are efficacious in minimizing health risks associated with both pesticide and nicotine exposure and that although the acute symptoms are similar for both pesticide and nicotine exposures, pesticide exposure additionally has long-term health risks.

### 5.2. Pesticide Training for Farmworkers

Training emerged as the most common facilitator of PPBs identified by farmworkers, and while documented problems with video training include lack of time for questions, the ability to look away and not engage with the video, and not meeting the needs of non-Spanish speakers [35], many farmworkers (*n* = 28, 39%) reported having learned from the video training or referred to the videos as a source of information. Very few farmworkers (*n* = 6, 8%) in this study reported receiving pesticide training in the form of a presentation/discussion.

Personal experience working in agriculture was described as a facilitator of PPBs, and less-experienced workers reported looking to those with more experience for guidance. However, other studies have reported that farmworkers who have been working in agriculture for a long time (8–10 years or longer) are less likely to use PPBs ([10,18]). Behavior modeling and discussions led by peer trainers who are moderately experienced (e.g., 4–8 years) in agricultural work might be an effective way of reinforcing what is shown in videos [36]. An experienced peer in the field who receives additional pesticide education may fill the need for an on-the-job safety resource, as prior studies have reported that farmworkers do not have anyone to ask about pesticides once they are working on farms [10]. Farmworkers who had worked for multiple seasons in the United States also described improvements in the work environment over time, which is consistent with previous reports of the perceptions of farm owners [26]. Findings from this study suggest that it would be helpful to implement discussion-based training led by moderately experienced peer trainers that takes into account the prior experience of farmworkers in both the United States and Mexico and that acknowledges safety advancements, while highlighting the continued need for individual protective behaviors and vigilance. These findings about what would make training more effective come at a crucial time when annual training is required as of 2017 [8] and new training materials are being developed.

### 5.3. Barriers to Protective Behaviors

Based on previous studies, we expected availability of supplies (or lack thereof) [12,13,14,15], time (or lack thereof) [10,11,12], and (dis)comfort [10,11,12,13] to be the most commonly cited barriers to PPBs. Previously, pesticide educators have also described time and weather as barriers [37]. These barriers, however, were less prevalent in this study. Another divergence from existing literature is the fact that farmworkers in this study never described a lack of washing supplies (such as soap and water) as a barrier to their washing behaviors, even though a complementary observational study demonstrated that washing supplies were unavailable in 33% of observations [18]. The most frequently described barrier to the use of PPBs by farmworkers was wetness. In a prior ethnographic study, farmworkers described their concerns about health problems—to include weak bones, joint aches, pneumonia, allergies, and skin irritation and itching—as being associated with being wet at work from irrigation water [38]. Dermal exposure is the most significant route of exposure for agricultural workers [39], and once clothing becomes wet with rainwater, dew, or sweat, it no longer provides adequate protection and may, in fact, increase absorption for both pesticides and nicotine [40]. 

### 5.4. Promising and Problematic Strategies from the Field

Farmworkers were much more likely to mention protective clothing than washing behaviors when discussing PPBs. When asked about PPBs, they identified clothing behaviors before washing behaviors, and sometimes did not comment on washing behaviors at all. In a complementary observational study, we found far greater adherence with protective clothing behaviors among farmworkers than with washing behaviors before eating and drinking in the field [18]. Findings suggest that farmworkers may undervalue the role of washing behaviors in minimizing exposure to pesticides in the field. PPB training should be reviewed to determine how hand-washing behaviors are presented and to explore ways to reinforce the importance of washing as a protective behavior to minimize exposure to pesticides (and nicotine) in the field.

Farmworkers identified changing clothing, with space and time to change provided by employers, as an important strategy to address wetness in the field, and they described utilizing techniques to overcome the obstacle of lack of clothing-change breaks, such as changing on the lunch break and in the bus between fields, as well as packing a change of clothes in backpacks left on the bus. Changing clothes at mid-day likely represents not only a reduction in wetness but also in pesticide and nicotine exposure [40,41].

During a complementary observational study, farmworker participants were observed wearing trash bags, thin ponchos, or light-weight rain jackets over their clothes while working in the fields [18]. In this study, farmworkers described these types of clothing as reducing wetness. There is limited literature evaluating these strategies for minimizing exposures to pesticides or nicotine [42]. Further, this observation raises concerns about increased risk for heat illness as recommendations related to additional opportunities for water, rest, and shade for workers wearing water-resistant clothing [40] were not implemented during field observations. Future interventions with farmworkers should address the dermal route of exposure as well as clothing behaviors that are adopted as protective by farmworkers but may actually be harmful, such as the use of a bandana to cover the nose/mouth, which may increase exposure through dermal and ingestion routes.

Farmworkers described medications and ointments as being beneficial in minimizing exposures. This finding was concerning because medications and ointments are not protective and at best treat symptoms that arise as a result of exposure. The prophylactic use of anti-itch medication and “suero,” powdered electrolytes brought from Mexico intended to treat dehydration caused by diarrhea, might actually be harmful if not properly utilized. More study on “suero,” its components, and its effects is needed. Additionally, drinking milk prophylactically—a strategy farmworkers sometimes use to feel better after exposure to nicotine [43] and as prevention [33]—has no proven efficacy in neutralizing pesticides other than in acute poisoning scenarios [44] and has no established benefit for routes of exposure other than ingestion. 

### 5.5. Limitations

The most significant limitation of this study is its inclusion of only farms that had previously participated in the voluntary Certified Safe Farm program. Certified Safe Farms were selected for participant recruitment because there was a complementary observational component to this study. In order to gain access to the private properties on which these farms were located and on which farmworkers were working, it was critical to obtain farm owner/operator consent so that observations could be conducted. While it might be expected that individuals operating Certified Safe Farms might be most likely to consent to observation on their farms, it is noteworthy that fewer than 50% of those approached for study participation consented to participate. Certified Safe Farms, by their nature, are likely to be more safety-oriented and to have a higher probability of providing resources and training to their workers than an average farm. Despite the fact that these farms likely represent a ‘best case scenario’ in supply provision and training, opportunities to improve farmworker safety and health on these farms still emerged. 

Other limitations to the generalizability of this study are its small number of farms, single crop, and single geographic location. The small number of farms and the variability in number of workers employed at each made it impossible to view the farms as independent units for analysis. The use of a structured interview guide and trained interviewer limited deeper exploration of some emerging beliefs and practices. 

Furthermore, the HBM has been criticized for failing to perform well with rural, underserved populations like this one [45]; other models, such as the Vulnerable Populations Conceptual Model, suggest that research consider resource availability, relative risk, and existing health status, which are pertinent to health promotion behaviors but not part of the HBM. 

Despite its limitations, this study was novel in its exploration of the modifying factors influencing PPBs. While barriers to and benefits of protective behaviors have been explored with farmworkers previously, this was the first examination of facilitators of PPBs and elicitation of strategies to improve PPBs. Farmworkers offered their perspectives on several novel protective behaviors that they had adopted. It was apparent that they were aware of short-term health risks associated with pesticide exposure and had adopted a variety of strategies, both those addressed in the WPS and some that are not, to mitigate exposure. However, they did not emphasize washing behaviors as playing an important role in PPBs. 

## 6. Conclusions

Within the context of the HBM, this analysis demonstrated that perceived benefits and perceived barriers of PPBs were insufficient to understand farmworkers' beliefs about PPBs and the PPBs practiced in the field. Likewise, an examination of perceived threat (severity and susceptibility) that we undertook was insufficient to predict PPBs [46]. In both what we observed [18] and what we heard, a host of other factors emerged that deserve additional consideration—peers as influencers of behavior, varying workplace safety climates, and farmworkers’ higher priorities than doing PPBs. Additionally, the emergence of training as a facilitator of PPBs and the value of peer leaders in the field as influencers of PPBs suggest that cues to action, such as advice from peers, should be specifically studied in the future and that educational interventions should incorporate these influential peers. 

Future research should focus on determining the efficacy and feasibility of farmworker-identified strategies in minimizing exposure to pesticides. Resulting data should be shared with policy makers, who may reconsider PPBs currently mandated to be taught and how and by whom protective clothing and supplies should be provided. In light of the fact that recent changes to the WPS are currently being implemented and new training materials are under development across the country, resulting data should also be shared with those who are revising and creating educational materials to reflect the updated WPS requirements. Based on the findings of the present study, new training materials should focus on farmworker understanding of short- vs. long-term risks posed by agricultural work, washing behaviors, and the benefits of protective behaviors to both mitigate nicotine and pesticide exposures. Another consideration for pesticide education developers is the establishment of programs to prepare moderately experienced farmworkers to become trainers who might support increased adherence to recommended behaviors and promote peer support in the field.

## Figures and Tables

**Table 1:** Personal Characteristics of Farmworkers.

Personal Characteristics	Mean (SD)	Frequency (%)
Demographics		
Age	32.8 (11.5)	
Gender		
Male		69 (96%)
Female		3 (4%)
Marital Status		
Married		36 (50%)
Civil union		20 (28%)
Not married		16 (22%)
Ethnicity: Latino		72 (100%)
Home Country		
Mexico		70 (97%)
Honduras		2 (3%)
Agricultural Experience		
Seasons lived in the United States	6.5 (5.6)	
Years worked in agriculture outside of United States	12.3 (10.3)	
Years worked in agriculture in the United States	6.4 (5.62)	
Years worked in tobacco	7.0 (5.6)	
In the United States on an H2A visa		
Yes		65 (90%)
No		7 (10%)
Traveled to another farm for agricultural work in last 12 months		
Yes		7 (10%)
No		65 (90%)
Live on the farm where you work		
Yes		69 (96%)
No		3 (4%)
Highest Level of Education Completed		
Less than middle school (grades 1–6)		26 (36%)
Middle school (grades 7–9)		38 (53%)
Some higher education or beyond (grades 10–12+)		8 (11%)
English Proficiency		
Skill in reading English		
None or very little		66 (92%)
Some		6 (8%)
Skill in writing English		
None or very little		69 (96%)
Some		3 (4%)
Pesticide Training		
Type of training (more than 1 response allowed)		
None		2 (3%)
Video		68 (94%)
Presentation/discussion		6 (8%)
Practice session		7 (10%)
Year of last pesticide safety training		
Never		2 (3%)
1–2 years prior		12 (17%)
Less than 1 year prior		58 (80%)
